# Vitamin E (Alpha-Tocopherol) Metabolism and Nutrition in Chronic Kidney Disease

**DOI:** 10.3390/antiox11050989

**Published:** 2022-05-18

**Authors:** Francesco Galli, Mario Bonomini, Desirée Bartolini, Linda Zatini, Gianpaolo Reboldi, Giada Marcantonini, Giorgio Gentile, Vittorio Sirolli, Natalia Di Pietro

**Affiliations:** 1 Department of Pharmaceutical Science, University of Perugia, 06126 Perugia, Italy; desiree.bartolini@unipg.it (D.B.); linda.zatini@studenti.unipg.it (L.Z.); giadamarcantonini94@gmail.com (G.M.); 2 Department of Medicine and Aging, G. d’Annunzio University Chieti-Pescara, 66100 Chieti, Italy; mario.bonomini@unich.it (M.B.); vsirolli@unich.it (V.S.); 3 Department of Medicine and Surgery, Centro di Ricerca Clinica e Traslazionale, CERICLET, University of Perugia, 06126 Perugia, Italy; paolo.reboldi@unipg.it; 4 Royal Cornwall Hospitals, NHS Trust, Cornwall, Truro TR1 3LJ, UK; g.gentile2@exter.ac.uk; 5 Department of Nephrology, University of Exeter Medical School, Exeter EX1 2HZ, UK; 6 Department of Medical, Oral and Biotechnological Sciences, Center for Advanced Studies and Technology-CAST, G. d’Annunzio University Chieti-Pescara, 66100 Chieti, Italy; natalia.dipietro@unich.it

**Keywords:** vitamin E, tocopherol, tocotrienol, lipidomics, metabolomics, renal disease, hemodialysis, membranes, lipid metabolism, antioxidants

## Abstract

Vitamin E (alpha-tocopherol) is an essential micronutrient and fat-soluble antioxidant with proposed role in protecting tissues from uncontrolled lipid peroxidation. This vitamin has also important protein function and gene modulation effects. The metabolism of vitamin E depends on hepatic binding proteins that selectively retain food alpha-tocopherol for incorporation into nascent VLDL and tissue distribution together with esterified cholesterol and triglycerides. Chronic kidney disease (CKD) is a condition of oxidative stress and increased lipid peroxidation, that are associated with alterations of alpha-tocopherol metabolism and function. Specific changes have been reported for the levels of its enzymatic metabolites, including both short-chain and long-chain metabolites, the latter being endowed with regulatory functions on enzymatic and gene expression processes important for the metabolism of lipids and xenobiotics detoxification, as well as for the control of immune and inflammatory processes. Vitamin E therapy has been investigated in CKD using both oral vitamin E protocols and vitamin E-coated hemodialyzers, showing promising results in the secondary prevention of cardiovascular disease, as well as of immune and hematological complications. These therapeutic approaches are reviewed in the present article, together with a narrative excursus on the main findings indicating CKD as a condition of relative deficiency and impaired metabolism of vitamin E.

## 1. Introduction

**Figure 1:**
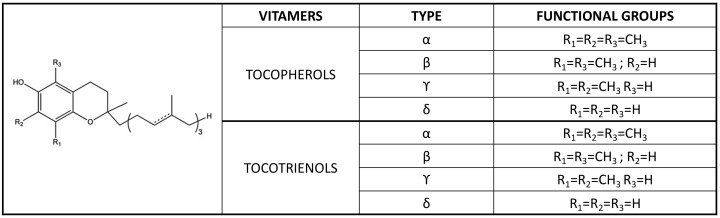
Structure and systematics of the main tocopherol and tocotrienol forms. These two subgroups of vitamers are characterized by an aliphatic phytyl side chain and an unsaturated farnesyl side chain, respectively.

Vitamin E was first isolated from the unsaponifiable lipid fraction of vegetable ingredients of animal diets that were discovered exactly one century ago to be essential to rat fertility [1,2]. The first vitamin structure to be identified was that of α-tocopherol (α-TOH) [3], but this vitamin is often described as a family of plant-derived molecules, or tocochromanols. These include eight fat-soluble vitamers, four tocopherols (TOH) and four tocotrienols (T3) (Figure 1) found in seeds, edible oils, and, in general, in dietary fats [4]. However, many other plant analogues are present in nature and some of them are now gaining increasing interest as plant medicinal compounds and nutraceuticals [5].

Alpha-tocopherol is the most abundant vitamin form found in human tissues and the form with higher biological activity in either the rat fertility test or other activity tests (reviewed in [4]). However, only α-TOH should deserve the definition of vitamin, i.e., of essential micronutrient [6]. In fact, only this tocochromanol appears to have the ability to reverse the neurological symptoms of an inherited form of ataxia associated with vitamin E deficiency (AVED) [7,8]; moreover, secondary deficiency states have been described for this vitamin form, including malabsorption, liver failure, and digestive tract diseases [4,9].

However, 100 years of scientific efforts have not been sufficient to define the exact molecular mechanism(s) responsible for the essentiality and for the other biological effects attributed to this vitamin (discussed elsewhere in [10,11] and references therein). The antioxidant activity has long been considered the main mechanism behind the biological properties of vitamin E [12]. In fact, since the pioneering studies leading to its discovery as an “antioxygenic” factor of wheat germ oil, this function was interpreted as crucial to prevent food lipid autoxidation [13,14]; this has been by far the most impacting evidence obtained on this vitamin that still today represents the most popular and marketable fat-soluble antioxidant ever; in fact, more than 70 Ktons of α-TOH are produced every year worldwide [15] for disparate industrial applications, including the lipid preservation of food, cosmetics, and pharmaceutical products, as well as the production of nutritional products, supplements and nutraceuticals.

Besides the antioxidant properties, α-TOH presents “non-antioxidant” functions [6,16] consisting of direct (physical) interactions and modulation of cellular proteins with enzymatic activity and transcriptional function on different groups of genes involved in important cellular processes, such as cell cycle regulation, proliferation and cell death signaling, antioxidant defense and detoxification of xenobiotics, inflammation, and response to the host (recently reviewed in [17,18]). In this respect, α-TOH appears to differ from the other vitamers and analogues owing to specific molecular interactions and effects on cellular pathways [19], some of which are summarized in Table 1.

The metabolism and tissue distribution of this vitamin are closely associated with those of cholesterol and thus of lipoproteins, involving both the TG-rich particles and the HDL fraction [4]. These are highly regulated processes that appear to depend on specific hepatic binding proteins, and especially of α-tocopherol transfer protein (α-TTP) (reviewed in [18,39,40]). This protein plays an important role in the interaction of this vitamin with both triglyceride metabolism and membrane phospholipids of the liver and other tissues [12,41,42,43], and mutations in the gene encoding for this protein are responsible of AVED [44,45,46].

**Figure 2:**
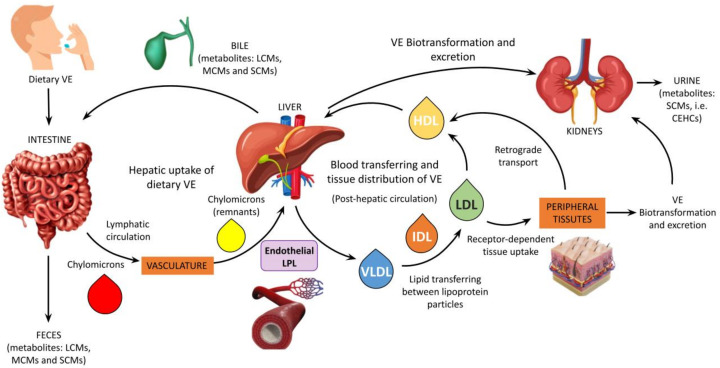
Schematic representation of vitamin E metabolism. The main steps of the intestinal, hepatic, and post-hepatic metabolism of vitamin E are shown. Those in the liver include cellular uptake, subcellular trafficking, biotransformation, incorporation into VLDL particles and excretion for tissue distribution. LCMs: long-chain-metabolites; MCMs: multi-cycling metabolites; SCMs: short-chain-metabolites; CEHCs: carboxyethyl hydroxychromans (natural vitamin E metabolites); VE: vitamin E; LPL: lipoprotein lipase.

Key steps of the hepatic metabolism of vitamin E include cellular uptake, subcellular trafficking, incorporation into VLDL particles or biotransformation, and excretion for tissue distribution (Figure 2).

Extrahepatic processes are also important to define the metabolism and nutritional status of this vitamin and include exchange of the vitamin between circulating lipoproteins, tissue uptake mechanisms by means of Apo-proteins and lipoprotein receptor function, cellular detoxification (which occurs by means of the same enzymatic machinery of the liver cell), and HDL-mediated reverse transport. None of these aspects are discussed in detail here since they lie beyond the scope of this article (for more details the reader can refer to the following review articles and book chapters [4,39,47,48,49,50]).

The biotransformation process of vitamin E [51] was originally identified as the catabolic pathway to degrade and excrete the excess of vitamin analogues introduced with the diet or present in the endogenous pool of vitamin E; however, as of the early 2000s, early evidence was obtained on discrete biological functions of some metabolites, suggesting a bioactivation role for this metabolic step [51], which is now supported by more robust evidence [49,52]. In fact, multiple roles have been identified for the long-chain metabolites of both α-TOH and the other analogues that are formed by enzymatic and free radical-dependent processes. These roles include the modulation of inflammatory and detoxification pathways of different cell models and tissues, as well as of lipid metabolism (recently reviewed in [53]). These biological effects depend on regulatory interactions with transcriptional and enzymatic proteins, some of which are summarized in Table 1, and may suggest nutraceutical or pharmacological applications for the different vitamers and LCMs analogues, such as the plant metabolite garcinoic acid, including lipid lowering and cardiovascular protection, anticancer, anti-inflammatory and detoxification effects of the GI tract and even of the brain [24,32,33,36,49,53,54,55,56,57,58]. 

In this respect, the activity of the LCMs derived from α-TOH and the extent of metabolic transformation of this vitamer, are markedly different from those of all other vitamers and vitamin analogues [49,55], once again suggesting the need for a revision of vitamin E nomenclature [6] to distinguish between the essential features of α-TOH and other properties of other analogues including their LCMs.

Chronic kidney disease (CKD) is a condition affecting an increased number of people with a major impact on health systems in both industrialized and developing countries [59]. As the disease evolves through various stages of severity, the patients develop an increased risk of oxidative stress and accelerated aging [60], as well as lipid abnormalities and malnutrition that may interfere with the metabolism and function of fat-soluble vitamins, including vitamins K [61,62], D [63], and E [64,65].

In the case of vitamin E, the pro-oxidant environment generated by the impaired renal function may entail an increased demand for this antioxidant vitamin to meet a higher defense burden of lipoproteins and sufficient cellular protection against lipid peroxidation. This is a chain reaction process that can cause lipoprotein and cellular membrane damage, and vitamin E is one of the physiological players that can intervene as chain breaker to scavenge lipoperoxyl radicals by its H atom donating properties [12,66]. Lipid peroxidation is considered a major event in lipotoxicity, i.e., the condition of toxicity observed in various tissues by the excessive or abnormal activity of lipids and their metabolites introduced with the diet or synthesized through endogenous lipogenesis pathways [67]. Such condition—primarily investigated in metabolic diseases, including obesity and NAFLD [68]—also affects CKD where a significant percentage of patients present hyperlipidemia and increased levels of free fatty acids [69,70], increased indices of oxidative stress and inflammation, the severity of which depends on adiposity [71], including modifications of polyunsaturated fatty acids (PUFA) of the ω-3 and ω-6 series that play a key role in the modulation of inflammatory processes [72,73]. In this scenario —as introduced earlier— a relative deficiency of vitamin E has consistently been shown to occur in CKD, which is worth investigating for its possible causal role in this clinical model of oxidative stress and lipotoxicity.

In this narrative review article, we describe the defects of vitamin E metabolism and function in CKD, including the most recent strategies and nutrigenomics tools available to characterize them at a clinical level and devise nutritional interventions and treatments, which include oral supplements and the use of vitamin E-coated dialyzers in end-stage renal disease patients (ESRD) on hemodialysis therapy (HD).

## 2. Disease-Specific Changes to Vitamin E Status and Metabolism in CKD

### 2.1. Vitamin E Deficiency and Lipotoxicity in CKD

CKD is a low-grade inflammation and oxidative stress condition [60,74,75,76]. This pro-oxidant environment is sustained by the toxicity and degenerative effects of uremic retention solutes, and by a defective renal metabolism. The latter plays a major role in inter-organ processes of redox-homeostasis and detoxification, including for example the metabolism of cysteine-glutathione system ([77,78,79] and references therein). Under these circumstances, increased levels of lipid peroxidation have consistently been demonstrated in blood cells and plasma of CKD patients, suggesting a defective protection of polyunsaturated fatty acids and cholesterol from the pro-oxidant effects of the uremic environment [64,80,81,82,83]. Moreover, oxidative stress and lipid peroxidation can be amplified by increased adiposity in moderate to severe CKD [71], that may lead to develop lipotoxicity and metabolic complications associated with hepatic and peripheral insulin resistance [67]. These complications also include dyslipidemia; hypertriglyceridemia (HTG) is highly prevalent in these patients, and although HTG per se does not appear to represent a risk factor for atherosclerotic cardiovascular disease [84], it can combine with mild to moderate hypercholesterolemia and with other—hitherto unidentified—factors to cause pro-atherogenic changes in circulating lipoproteins, such as the formation of small-dense LDL particles and/or defects in HDL sub-fractions [51,67,68,69]. Such pro-atherogenic changes have long been conjectured to include a defect in the antioxidant systems involved in preventing lipid peroxidation and apoprotein damage. In this respect, vitamin E and selenium play a key role in the interplay between enzymatic and non-enzymatic pathways that prevent lipid peroxidation of circulating lipoproteins as well as of cellular membranes [4,11,85]. Changes in dietary habits during patient management and the presence of secondary malnutrition consequent to CKD comorbidity, are both capable of interfering with the intake of antioxidant micronutrients and fat-soluble vitamins, including vitamin E [65].

Accordingly, after correction for blood lipids, plasma concentrations of α-TOH can be significantly lower in CKD and ESRD patients on regular HD than in healthy controls [64,65]. Furthermore, exposing total plasma or the LDL fraction of CKD patients to a pro-oxidant insult results in a higher consumption of vitamin E compared to controls [69,85,86], pointing out the increased susceptibility of plasma lipids to oxidative damage in this disease [82,83]; such susceptibility and the defective antioxidant protection of tissue lipids in CKD is confirmed by the increased decoration of plasma proteins by reactive carbonyls deriving from lipid peroxidation, also known as advanced lipoxidation end-products (ALEs) [87,88].

**Figure 3:**
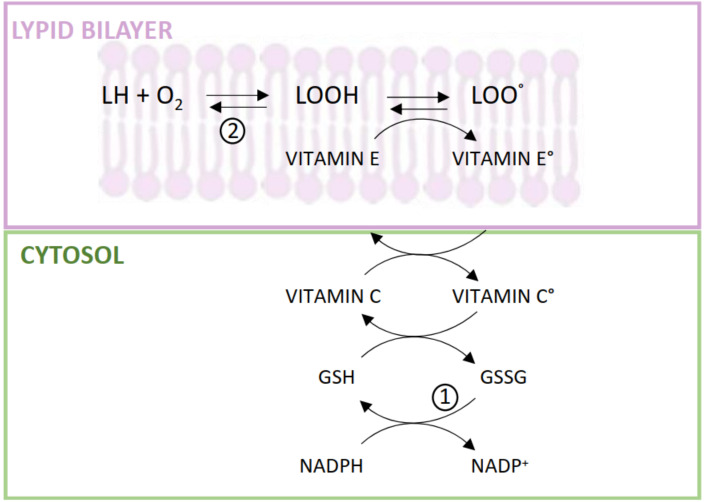
Interaction between hydrophilic antioxidants and vitamin E in the scavenging of lipoperoxyl radicals formed in the cellular membrane. Abbreviations: L, membrane lipids; Vitamin E°, tocopheryl radical; Vitamin C°, ascorbyl radical; GSH and GSSG, reduced and oxidized glutathione; NADP+ and NADPH, nicotinamide-adenine-dinucleotide phosphate oxidized and reduced-form. (1) Hexose monophosphate shunt and GSH-reductase activity; (2) Membrane GSH-peroxidase activity.

Defects in the co-antioxidants involved in restoring the vitamin E redox during lipid peroxidation (Figure 3) may also contribute a role in this context [89]. For example, the antioxidant function of vitamin E could be lower than normal in CKD, and especially in HD patients, secondarily to a defective vitamin C status or redox balance (the importance of this vitamin as co-antioxidant of vitamin E has recently been reviewed in [90]); in fact, this is a water-soluble antioxidant undergoing chronic leakage and increased oxidation to ascorbyl radical during dialysis therapy [65,91,92].

These aspects reveal a condition of relative deficiency of vitamin E in CKD, i.e., relative to other lipid molecules present in circulating lipoproteins and to the demand for antioxidant protection and redox hemostasis of uremic tissues. By way of confirming the importance of this relative deficiency of vitamin E as a cause of defective protection of tissue lipids, vitamin E therapy has been shown to partially relieve lipid peroxidation and some inflammatory and oxidative-stress complications of CKD (presented later in Section 2.3).

### 2.2. The Vitamin E Metabolome and Its Alterations in CKD

Recent advancements in metabolomics and lipidomics technology have provided an opportunity to develop methodologically unbiased protocols designed to characterize the human metabolism of vitamin E at different levels of vitamin intake and in the presence of specific diseases, including CKD [73,93,94].

This metabolome includes short- (SCM), middle- (MCM), and long-chain metabolites (LCMs) formed during the enzymatic biotransformation of this vitamin, as well as SCMs and LCMs deriving from free radical-dependent transformation (i.e., Simon metabolites and tocopheryl quinone or TQ, respectively, formed by the vitamin’s antioxidant activity) (Figure 1) [95,96].

CKD presents characteristic changes in the enzymatic metabolism of vitamin E that are associated with a disproportion between the molar ratio of α-TOH and circulating lipids (described in Section 2.1). The first evidence of such an alteration came in the early 2000s when some of us and other authors [65,97] found CKD and ESRD-HD patients to have markedly increased plasma levels of the SCM 2(2′-carboxyethyl)-6-hydroxychroman (CEHC) formed during the catabolism of the main forms of vitamin E found in human tissues, i.e., α-TOH and γ-TOH (Figure 1). SCMs are the end-products of CYP450-mediated activation of the vitamin side-chain catabolism that proceeds through a β-oxidation-like mechanism to decrease vitamer hydrophobicity and facilitate chromanol ring excretion through the bile and to a lower extent through urine after phase 2 derivatization of the carboxylic group with sulphate or glucuronide residues [51,98]. Hence, the increased levels of SCMs in CKD patients (Table 2) stem from reduced renal clearance of these water-soluble compounds, which represents a major confounder for the use of these metabolites as indicators of vitamin E adequacy even in the healthy [99,100,101]. However, these enzymatic products can be measured in plasma as a reliable indicator of vitamin E biotransformation in the human liver [101], and recent standardization of the assay procedure by GC- or LC-MS/MS has demonstrated the reliability of this assay as a way of also assessing the response to vitamin supplementation [94,95].

The levels of these plasma SCMs linearly follow the reduction of GFR in the different stages of CKD [65], and can reach up to 10 or 20-fold higher levels in ESRD patients than in healthy subjects [93] (Table 2).

The levels of long-chain metabolites (LCMs) are independent of renal excretion efficiency, these being much more lipophilic than SCMs. Human blood concentrations of these metabolites are in the low nanomolar range [52,93,96] and are almost exclusively affected by the activity of enzymatic and transport proteins involved in hepatic biotransformation. They nonetheless likewise present characteristic changes (summarized in Table 2) that have now been determined with the validated metabolomics platform utilized to assess the human metabolome of vitamin E [73,94].

By way of greater detail, data presented in Table 2 describe how CKD patients show reduced plasma concentrations α-13′-OH (Figure 1) compared with healthy controls. Decreased levels were also observed for M1, another unknown LCM; this metabolite, first discovered during vitamin E supplementation in humans, presents as an isobaric and isomeric form of α-13′-OH [93]. Moreover, reduced levels of PUFA metabolite 20-COOH-AA were also observed in CKD patients; interestingly enough is that this enzymatic oxidation metabolite of AA is produced by the same CYP450-mediated ω-oxidation process that forms α-13′-COOH, and significant correlations between its levels and the levels of the LCMs M1 and M3 were observed in healthy controls (R2 = 0.259; *p* = 0.013 and R2 = 0.309, *p* = 0.006, respectively), while CKD patients showed such correlation only for the LCM M3, thus confirming a defect in the ω-oxidation pathway of AA and LCMS in these patients.

Again, CKD patients showed reduced levels of DHA (Table 2), which confirms a defective ω-3 fatty acids metabolism in these patients [72] with resulting increase of AA/DHA ratio. Together with the observed trend toward an increase in α-TQ concentrations, these changes in DHA and AA/DHA levels point out an increased risk of inflammation and lipotoxicity [53].

To sum up, the alterations of the vitamin E metabolome in CKD patients show (1) a defective processing of vitamin E throughout the earliest steps of its enzymatic metabolism, as demonstrated by the markedly reduced levels of the LCMs α-13′-OH and M1 (with the latter decreasing approximately 20 fold *vs*. mean control levels); and (2) increased vitamin oxidation through the free radical-dependent pathway that was associated with lipotoxicity and a pro-inflammatory phenotype due to the altered metabolism of the ω-6 AA and the ω-3 DHA.

### 2.3. Vitamin E Supplementation and Therapy in CKD

#### 2.3.1. Oral Supplements

The defects in the vitamin E metabolism and function described earlier in Section 2.1, support the notion that a higher intake of vitamin E in CKD and ESRD patients undergoing HD, may help to restore the vitamin E/lipid ratio found in healthy subjects or even increase it to higher levels in order to increase the antioxidant protection of the tissues [64]. Accord with this latter option, HD patients treated with vitamin E showed increased ex vivo antioxidant protection in their plasma LDL fraction [82], while increased levels of vitamin E in leukocytes and red blood cells of patients treated with vitamin E-coated hemodialysers were associated with higher protection against lipid peroxidation, functional damage and cell death, caused by the poor biocompatibility and pro-inflammatory effects of HD therapy [89,102].

Pre-clinical evidence obtained in animal models of kidney disease and some observational and small intervention studies suggest that CKD and ESRD could be among the conditions of increased cardiovascular risk that are likely to benefit from vitamin E supplementation. However, there is no clear-cut evidence that vitamin E supplementation can either extend life expectancy or reduce the risk of cardiovascular complications in non-dialysis CKD patients. In a post hoc analysis of the Heart Outcomes Prevention Evaluation (HOPE) trial, 993 patients with mild to moderate CKD (as defined by serum creatinine ≥ 1.4 up to 2.3 mg/dL) were given either 400 IU of oral vitamin E daily or placebo. Over a median follow-up time of 4.5 years, there was no difference between the two groups in terms of all-cause mortality, cardiovascular mortality, transient ischemic attack, stroke, myocardial infarction, or heart failure [103]. The effects of vitamin E supplementation on anthropometric measures and inflammatory markers were investigated by Ramos and coworkers in 62 patients with stage 3 or 4 CKD randomized to either mixed tocopherols plus alpha lipoic acid (666 IU/daily and 600 mg daily, respectively) or placebo [104]. After two months of follow-up, there was no beneficial effect from vitamin E supplementation versus placebo on BMI, body weight, or several inflammatory markers, including interleukin-6, C-reactive protein, F2-isoprostanes, or protein thiols. Likewise, it is currently unclear if vitamin E supplementation can slow down the decline in kidney function over time. Finally, vitamin E does not provide any benefits in the prevention of contrast-dye nephropathy in patients with underlying CKD [105]. On the other hand, some positive effects of vitamin E supplementation have been reported in diabetic kidney disease (DKD) patients. In a recent meta-analysis of RCTs carried out in Type 1 and Type 2 patients, Bolignano et al. [106] reported that vitamin E administered alone or in combination with other antioxidants might ameliorate albumin excretion, but not eGFR, though this needs to be further verified in less heterogeneous studies. Interestingly, the reverse effects (amelioration of eGFR but not albumin excretion) have been observed with tocotrienol-rich vitamin E in a recent phase IIb randomized, controlled, double-blind, placebo-controlled trial in 59 patients with DKD and stage 3 CKD followed up for a period of 12 months. Specifically, vitamin E supplementation improved eGFR compared to placebo at eight months (mean difference 1.9 ± 5.76 vs. −3.29 ± 9.24; *p* = 0.011), but those benefits were not maintained at 12 months [107] and were not paralleled by any benefits to albuminuria or serum biomarkers, including vascular endothelial growth factor (VEGF) or transforming growth factor beta 1 (TGF-β1)—a fibrinogenic cytokine that plays a key pathogenetic role in DKD. Key limitations of this study are the relatively small sample size and the lack of inclusion of additional DKD biomarkers. Further trials are needed, aimed at assessing the comparative efficacy of tocotrienols versus tocopherols in DKD patients. Beneficial effects on renal parameters of diabetic nephropathy have been observed in other intervention studies and preclinical models that have recently been reviewed by Di Vincenzo et al. [108], highlighting the importance of vitamin E in controlling cardiometabolic and inflammatory complications of diabetes, as well as gene polymorphisms such as the haptoglobin 2-2 genotype (Hp 2-2) which is emerging as a critical variable for the efficacy of antioxidant therapy as well as for the development of renal complications and for CVD risk [109]. In this population, the administration of vitamin E might offer a low-cost and accessible means of reducing CV events and mortality that is worth investigating.

In the case of oral supplementation protocols for this vitamin (i.e., α-TOH), particular care should be paid to avoid depletion of non-vitamin homologues, and particularly γ-TOH, which can undergo higher metabolization by supplement-induced activation of detoxification pathways, especially the hepatic PXR/CYP450 system [94]. γ-TOH is reported to have antioxidant and anti-inflammatory roles that may synergize and even implement those of α-TOH and its bioactive metabolites [22,110,111].

To sum up, considering the limited number of high-quality trials in pre-dialysis CKD, we would advocate adopting the position of the recent KDOQI Clinical Practice Guidelines for Nutrition in CKD (2020 update) [112], which suggest that there is insufficient evidence to make recommendations on vitamin E intake in CKD patients. Additionally, the prevalence of vitamin E deficiency in pre-dialysis CKD patients is unclear, and there is some concern regarding high-dose supplementation of vitamin E, given the potential increased risk of hemorrhagic stroke and impaired platelet aggregation, as well as interactions between vitamin E and other antiplatelet and anticoagulant medications. A tailored approach could be considered in individual patients, considering several parameters including the nutritional status and dietary intake, concomitant medications, etc., while oral doses ≥ 400 IU/daily should be avoided [112].

In patients on maintenance HD, the large RCT “Secondary prevention with antioxidants of cardiovascular disease in end-stage renal disease” study (SPACE) investigated the effect of pharmacological dosages of vitamin E on CV disease outcomes [113]. In this study, treatment of pre-existent CV disease patients with vitamin E (800 U/day) resulted in a significant reduction (54%) of the primary endpoint (a composite variable including myocardial infarction, ischemic stroke, peripheral vascular disease, and unstable angina) and a 70% reduction in myocardial infarction. However, the potential for vitamin E toxicity should always be taken into account in HD or peritoneal dialysis (PD) patients, and if supplementation is warranted, excessive doses should be avoided and the patient should be monitored for toxicity [112]. Two systematic reviews and meta-analyses have recently been conducted in HD patients treated with oral vitamin E. Bergin et al. [114] indicated that vitamin E supplementation in HD patients resulted in significantly lower levels of malondialdehyde (MDA), a biomarker of oxidative stress, though their pooled results from 16 studies did not show a comparison with control groups. Nguyen et al. [115] examined 11 trials with four hundred and ninety-one randomized patients. Their pooled data showed that, as compared to control groups, vitamin E supplementation significantly reduced biomarkers of vascular inflammation (intercellular adhesion molecule-1 and vascular cell adhesion molecule-1), oxidative stress (MDA), and inflammation (CRP, but not IL-6). Both these studies, however, suffer from some methodological shortcomings, particularly heterogeneity among the trials included, so that further RCTs of higher quality are needed before one can recommend oral vitamin E supplementation to patients on HD.

#### 2.3.2. Vitamin E-Coated Dialyzers

HD is a life-sustaining therapy for millions of people all over the world. In HD, the artificial membrane contained in the hemodialyzer represents the main determinant of the success and the quality of the extracorporeal therapy [116]. Besides removal of retained toxic substances and excess water accumulated in uremic blood because of kidney failure, interactions between the membrane material and blood components occurring during the HD procedure which characterize the membrane’s bio(in)compatibility have focused clinical interest [117,118]. Poor biocompatibility results in proinflammatory, prooxidant stress and prothrombotic events [119]. The development of more biocompatible polymers to be used as hemodialyzer membrane is a major challenge when it comes to improving results and clinical patient outcome [120].

Several new membrane materials have been developed over the years using physicochemical methods or surface biofunctionalization, in an attempt to minimize the unfavorable interactions with blood components [121,122,123]. The biofunctional methods of surface modification include coating of the surface with vitamin E. Bioactive vitamin E (alpha-tocopherol) present on the blood surface of the modified membrane acts as an ROS scavenger to control oxidative stress and lipid peroxidation [124]. Vitamin E–modified membrane was designed to reduce oxidative stress in HD patients and improve biocompatibility [118]. This membrane has been defined as “interactive or functional membrane”, extending the concept of biocompatibility (mainly better control of blood cell activation) to include that of antioxidant bioactivity which provides protection against oxidative reactions occurring during the HD procedure [125]. Antioxidant therapy offers a promising strategy to reduce CV risk in patients on maintenance HD [126].

In the early 1990s, a cellulose-based vitamin E-bonded dialyzer membrane was developed and introduced on the market by the Terumo Corporation, Japan, under the commercial name of Excebrane^TM^. The base membrane was regenerated cellulose hollow fiber coated on the blood surface with a two-layer synthetic copolymer that binds a film of oleic alcohol to which alpha-tocopherol is in turn bound through hydrophobic interaction. The original concept adopted by this type of membrane is to give specific and timely protection against ROS on the membrane surface, the main site in which they are produced. Reduction of oxidative stress and inflammation compared to a non-coated cuprammonium rayon membrane was reported by Galli et al. [64]. Other reports showed use of cellulose-based vitamin E-coated membrane to be associated with improved erythrocyte life span and rheology, reduction of anticoagulant dosage, suppression of leukocyte activation, improved biocompatibility, and decreased levels of advanced glycation end-products [119,127].

Later, polysulfone (PS)-based vitamin E-coated membranes were developed to achieve a synergistic effect on the biocompatibility of the synthetic polymer and the antioxidant activity of vitamin E. PS-based dialyzers, initially developed by the Terumo Corporation, are now being manufactured using a new technique by Asahi Kasei Kuraray Medical Co., Japan, under the commercial name of VitabranE^TM^. The backbone of this membrane is a composite PS-polyvinylpyrrolidone copolymer that has been developed to produce optimum flow dynamics and clearance rates [125]. Many studies were carried out in HD to examine the effects of vitamin E-coated PS membrane (ViE-m), mainly on oxidative stress, inflammation, and need for erythropoiesis-stimulating agents (ESAs). Despite a wealth of data, however, there remain some inconsistencies between clinical trials, leading to conflicting results.

The favorable effect of using PS membranes coated with vitamin E on oxidative stress during HD session has been consistently demonstrated. In 2017, a systematic review and meta-analysis including sixty studies [128] showed that ViE-m produced a significant improvement in sensitive clinical biomarkers of oxidative stress such as plasma and erythrocyte MDA, thiobarbituric acid reactive substances, and plasma and erythrocyte vitamin E levels. These effects may have clinical significance, since biomarkers of oxidative stress may be connected with mortality and morbidity in HD patients [129], while long-term evaluations have shown that lower MDA levels are associated with reduced mortality [130]. In a 1-year study in 25 HD patients treated with ViE-m, Calò et al. [131] reported a significant decrease in the expression of proteins and markers pertaining to oxidative stress that are closely associated with cardiovascular disease. Analysis of the carotid artery intima-media thickness, an indicator of atherosclerosis, showed a trend toward reduction, suggesting potential CV protection brought about by ViE-m use.

The antioxidant efficacy of ViE-m was further demonstrated in 29 HD patients switched from conventional membranes to the use of ViE membranes for 6 months [132]. The study investigated the high levels of genetic damage in HD patients, which have been correlated with oxidative stress [133,134]. DNA damage was measured using micronucleus and comet assays both before and after the follow-up period. By the end of the study period, vitamin E deficiency was corrected, and levels of oxidative DNA damage proved to be significantly decreased. The authors also highlighted a significant increase in the hemoglobin status [132]. Conversely, little influence on markers of oxidative damage was highlighted by Djuric et al. [134]. The authors tested ViE-m in subjects suffering from the lack of glutathione transferase M1 enzyme activity (GSTM1), a condition associated with increased oxidative stress and mortality [135]. Eighty HD patients were equally randomized to either high-flux conventional PS dialyzers or vitamin E-coated PS dialyzers for three months. No differences between the two groups were demonstrated regarding markers of protein and lipid oxidative damage, inflammation (thiol groups, MDA, interleukin-6) or plasma antioxidant activity (glutathione peroxidase and superoxide dismutase).

A meta-analysis by Yang et al. [136], while showing the positive effect of using ViE-m on the reduction of oxidative stress biomarkers, demonstrated an improvement in the inflammatory status, namely a significant decrease in interleukin-6 (IL-6) and C-reactive protein (CRP) levels. In a subsequent meta-analysis including a larger number of studies, the improvement in the inflammatory status was confirmed for IL-6 but not for CRP levels [112]. More recently, Sepe et al. [137] conducted a randomized controlled crossover trial in eighteen HD patients, each treated with low-flux PS hemodialysis, low-flux bicarbonate hemodialysis with vitamin E-loaded dialyzers, and hemodiafiltration. The authors investigated inflammaging, which is a low-grade, sterile, non-resolving inflammatory state found in chronic disorders [138]: for this they assessed indoleamine 2,3-dioxygenase-1 activity (IDO1)—a macrophage proinflammatory stimulator—and nitric oxide (NO) formation. These parameters, which are involved in innate and adaptive immune response and in promoting chronic inflammatory disorders [139,140], proved to be higher in HD patients than in healthy controls. During the six-month follow-up, it was found that the most biocompatible procedure was when using ViE-coated membrane, which resulted in a significant reduction of IDO1 activity and NO formation as compared to the other two treatments. The data also showed significantly reduced NO serum levels in samples drawn just after ViE-m surface contact, further confirming the improved biocompatibility of the modified membrane [137].

Biocompatibility of ViE-m was also studied in seventeen patients on HD [141], treated in a clinical study with conventional high-flux hemodialyzers for 2 weeks (Pre-Vie phase) and switched to the ViE-m PS hemodialyzer for 36 sessions (ViE phase), followed by an additional 2 weeks on conventional hemodialyzers (Post-ViE phase). ViE-m showed no detectable difference compared to conventional treatment on leukocyte and C3a fluctuations during the HD sessions, but induced more stability in the platelet count, suggesting lower activation on the part of the platelets. This might also imply a reduction in the need for anticoagulation use during dialysis therapy. Notably, in that study, three anticoagulation-free sessions were successfully performed using ViE-m [141]. Previous reports had shown that vitamin E-bonded PS hemodialyzers were successful in reducing the anticoagulation requirement in HD treatments [142] and were not inferior to heparin-coated hemodialyzers in preventing circuit-clotting events leading to premature ending of dialysis sessions (in a randomized crossover study [143]). Another interesting finding of the study was that the frequency of hypotensive events during the extracorporeal procedure was reduced by about 30% in the ViE-phase [141]. This finding might be related to the reduced oxidative stress induced by ViE-m and is in keeping with previous findings [144].

Several studies pertaining to the anemic status of HD patients have been performed with vitamin E-coated dialyzers. Vitamin E can protect erythrocyte membrane against peroxidation, thereby increasing red blood cell survival [102], and can reduce the levels of pro inflammatory cytokines that inhibit erythropoiesis. Anemia is a major and common complication in HD patients, usually treated with and responsive to ESA [145]. However, some patients display a poor response to ESA, a condition called ESA resistance. The main cause of hyporesponsiveness to ESA is the systemic inflammation of CKD, known as inflammation anemia, a key role being played by IL-6 [146]. ESA resistance may require higher doses of ESA which, however, may be associated with an increased cardiovascular risk [147].

Meta-analyses showed that ViE-m significantly reduced the ESA Resistance Index (ERI) [148]. This effect was subsequently confirmed in a cross-over study in HD patients being treated in 13 centers throughout Italy: they were randomized to either a ViE-coated PS dialyzer (*n* = 48) or to a low-flux synthetic dialyzer (*n* = 46) for 12 months [149]. Mean ERI decreased in the Vie group by 1.45 IU/kg and increased in the control group by 0.53 IU/kg (*p* = 0.001). This observation, possibly related to a decrease in the IL-6 level [149], may be of clinical relevance, as ESA-resistance is a major obstacle to improving anemia control in the HD population.

A new field of research has emerged that recognizes the importance of the bioactivity of HD membranes and focuses on making other structural modifications and using other bioactive compounds to positively influence inflammation and oxidative stress. As reported in a recent review [101], vitamin E-coated membranes have the advantages of decreasing oxidative stress, improving inflammatory status and, partially, anemia. Use of ViE-m can improve some HD associated parameters (ESA resistance, intradialytic hypotension, anticoagulation requirement) but does not influence others such as dialysis adequacy, lipid profile, and serum albumin [128,148]. The role of this promising membrane material in the overall management of patients on maintenance HD requires further adequately powered clinical trials.

## 3. Conclusions

In conclusion, this review article summarizes the evidence indicating how solute retention, and inflammatory and metabolic complications conspire to sustain oxidative stress and lipid peroxidation as main cellular processes standing behind the premature aging and degenerative transformation of uremic tissues. In this respect, oxidative stress has been demonstrated conspiring with uremic toxins and patient’s comorbidity to sustain an abnormal activation of stress response genes, including those involved in the biosynthesis and detoxification function of the cellular antioxidant glutathione ([79] and references therein), and the induction of cell death programs, including apoptosis and ferroptosis [150,151,152]. Other consequences of cellular oxidative stress and lipid peroxidation in uremia and dialysis patients include the induction of ER stress and cell senescence, as well as autophagy inhibition [153] sustaining the loss of cellular mass and physiological functions of uremic tissues [154,155,156]. These processes exemplify CKD as a clinical model of allostatic load and accelerated aging [60,157,158,159].

Defects in the intake, metabolism, and antioxidant function of vitamin E coexist with these alterations and may play their part in the premature aging of these patients, increasing the uremic tissues’ susceptibility to oxidative stress and abnormal lipid peroxidation. Consequently, the role of this vitamin as a nutraceutical and therapeutic agent controlling the allostatic load and the degenerative processes of tissues is worth investigating in CKD and more in general in the multiple defects of the uremic syndrome. At present there is insufficient evidence to recommend increasing the intake of vitamin E in CKD patients, whereas results obtained in RCTs performed so far in HD patients treated with oral vitamin E are consistent with improved biomarkers of oxidative stress inflammation and vascular damage. However, the quality of these studies is not sufficient to provide conclusive evidence as to the benefits of vitamin E supplementation in these patients and, thus, further clinical investigation is called for. Vitamin E-modified hemodialyzers are an application of this vitamin, alternative to oral supplementation, in the clinical management of uremic and hemodialysis-related complications. The advantage of coating dialysis membranes with vitamin E is that this molecule increases at the same time the biocompatibility of the biomaterials and the antioxidant protection of blood components [89]. RCTs on these membranes have demonstrated promising results with benefits to oxidative stress, inflammation, vascular damage, and clinical management of anemia (effects being reported on ESA resistance, intradialytic hypotension and anticoagulation requirement): in short, worth investigating in higher quality trials.

## Figures and Tables

**Table 1:** Some molecular targets and gene response systems under the influence of α-tocopherol (vitamin E) and its analogues and endogenous metabolites.

Biological Function	Tocopherol Effect (Up ↑ or Down ↓ Regulation)	Gene Systems	References
Immune-inflammatory response and ferroptotic signaling:	α-TOH acetate ↓	NFkB	[20,21]
	Non-alpha LCMs ↓ (and to a lower extent α-LCMs)	LOX-5 and COXs	[22,23,24]
	α-TOH ↓ (possibly by conversion to α-tocopheryl hydroquinone)	LOX-15	[25,26,27]
Cholesterol homeostasis	γ-TOH and α-TOH ↑	PPARγ	[28]
	α-TOH ↓	LXR (LXR-regulated receptors: CD36, ABCA1, ABCG1)	[29]
Lipid metabolism and lipotoxicity-related inflammation	γ and α-TOH ↑	PPARα	[30]
	Garcinoic acid and α-LCMs ↑	PPARγ	[31,32,33]
Steroidogenesis	α-TOH ↑	AP-1 and Ref-1	[34]
Platelet aggregation	α-TOH↓	GP IIb	[35]
Xenobiotic detoxification	T3 ↑ LCMs ↑ (particularly δ-T3-13COOH or garcinoic acid)	PXR (PXR-regulated genes: CYP3A4 Pgp)	[36,37]
Estrogen receptor	T3 ↑	Erβ (Erβ-regulated genes: MIC-1, ECR-1 and cathepsin D)	[38]

Abbreviations: Tocotrienols (T3); Tocopherols (TOH); Long-chain metabolites (LCMs); delta-tocotrienol-13′-carboxy LCM (δ-T3-13′COOH); Nuclear factor kappa-light-chain-enhancer of activated B cells (NFκB); Peroxisome proliferator-activated receptor gamma (PPARγ); Liver X Receptor (LXR); cluster of differentiation 36 (CD36); ATP-binding cassette transporter (ABCA1); ATP-binding cassette G1 transporter (ABCG1); Peroxisome proliferator-activated receptor alpha (PPARα); Activator protein-1 (AP-1); Redox-regulated protein (Ref-1); Glycoprotein IIb (GP IIP); Pregnane X receptor (PXR); Cytochrome P3A4 (CYP3A4); estrogen receptor beta (ERβ); Macrophage inhibitory cytokine 1 (MIC-1); Estrogen receptor 1 (ECR-1).

**Table 2:** Vitamin E and PUFA parameters in plasma of healthy control subjects and chronic kidney disease (CDK) patients measured by the developed LC-MS/MS method (23 observations).

Parameters	Healthy Controls		CDK Patients		
	Mean	SD	Mean	SD	*p* ^a^
α-TOH (nM)	31,014	6535	23,873	8381	*
α-TQ (nM)	180	73	266	151	Ns
α-13′-OH (nM)	3.9	1.6	1.4	0.93	**
M3 (nM)	1.8	1.5	2.7	1.1	Ns
Sum 13′-α-OH + M3 (nM)	4.8	1.3	4.1	1.6	Ns
α-13′-COOH (nM)	1.8	1.0	1.8	1.3	Ns
M1 (nM)	47.8	36.6	4.5	5.7	**
M2 (nM)	7.4	7.5	5.2	3.0	Ns
Sum α-13′-COOH + M1 + M2 (nM)	57.0	33.6	11.5	8.1	**
α-CEHC (nM)	25.5	12.6	288	241	*
ϒ-TOH (nM)	618	73	862	411	*
ϒ-CEHC (nM)	122	59	645	392	**
Sum α-LA + ϒ-LA (nM)	3184	1165	2851	3139	Ns
EPA (nM)	324	202	215	198	Ns
DHA (nM)	2363	501	1842	843	*
AA (nM)	3109	498	3532	1629	Ns
20-COOH-AA (nM)	21	16	8.2	8.9	**
20-COOH-AA/AA × 1000	7.8	4.4	2.3	2.1	**
EPA/DHA	0.11	0.07	0.10	0.05	Ns
AA/DHA	1.4	0.4	2.0	0.5	**

^a^ Kruskas-Wallis non-parametric test (*p*): * ≤ 0.05, ** *p* ≤ 0.01; ns = not significant. Data were from [74,95,96]. Abbreviations: α-tocopherol and g-tocopherol (α-TOH and g-TOH, respectively); α-tocopheryl quinone (α-TQ); α-13′-hydroxy and carboxy long-chain metabolites, (α-13′-OH and α-13′-COOH, respectively); unknown vitamin E LCMs (M1-3); 2, 5, 7, 8-tetramethyl-2-(2′-carboxyethyl)-6-hydroxychroman (alpha-CEHC); 2, 7, 8-trimethyl-2-(2′-carboxyethyl)-6-hydroxychroman (gamma-CEHC); alpha-linolenic acid (a-LA); gamma-linolenic acid (g-LA); Eicosapentaenoic Acid (EPA); Docosahexaenoic Acid (DHA); Arachidonic Acid (AA); 20-carboxy-AA (20-COOH-AA).
